# Effects of Amendments and Indigenous Microorganisms on the Growth and Cd and Pb Uptake of Coriander (*Coriandrum sativum* L.) in Heavy Metal-Contaminated Soils

**DOI:** 10.3390/toxics10080408

**Published:** 2022-07-22

**Authors:** Nana Mi, Wenying Hao, Zixin Zhou, Longcheng Li, Fayuan Wang, Jingping Gai

**Affiliations:** 1 Beijing Key Laboratory of Farmland Soil Pollution Prevention and Remediation, College of Resources and Environmental Sciences, China Agricultural University, Beijing 100193, China; minn2015@126.com (N.M.); haowying@163.com (W.H.); zzx2622818207@163.com (Z.Z.); lilongchengwy@163.com (L.L.); 2 Key Laboratory of Plant-Soil Interactions, Chinese Ministry of Education, College of Resources and Environmental Sciences, China Agricultural University, Beijing 100193, China; 3 College of Environment and Safety Engineering, Qingdao University of Science and Technology, Qingdao 266042, China

**Keywords:** heavy metal pollution, vegetable, nano-hydroxyapatite, poly γ-glutamic acid, soil microbe

## Abstract

Heavy metal (HM) contamination of soils is a worldwide problem with adverse consequences to the environment and human health. For the safe production of vegetables in contaminated soil, efficient soil amendments need to be applied such as nano-hydroxyapatite (n-HAP) and poly γ-glutamic acid (γ-PGA), which can mitigate heavy metal uptake and enhance crop yield. However, the combined effects of soil amendments and indigenous microorganisms (IMOs) on HMs immobilisation and accumulation by crops have received little attention. We established a pot experiment to investigate the effects of IMOs combined with n-HAP and γ-PGA on coriander (*Coriandrum sativum* L.) growth and its Cd and Pb uptake in two acidic soils contaminated with HMs. The study demonstrated that applying n-HAP, with and without IMOs, significantly increased shoot dry biomass and reduced plant Cd and Pb uptake and diethylenetriaminepentaacetic acid (DTPA) extractable Cd and Pb concentrations in most cases. However, γ-PGA, with and without IMOs, only reduced soil DTPA-extractable Pb concentrations in slightly contaminated soil with 0.29 mg/kg Cd and 50.9 mg/kg Pb. Regardless of amendments, IMOs independently increased shoot dry biomass and soil DTPA-extractable Cd concentrations in moderately contaminated soil with 1.08 mg/kg Cd and 100.0 mg/kg Pb. A synergistic effect was observed with a combined IMOs and n-HAP treatment, where DTPA-extractable Cd and Pb concentrations decreased in slightly contaminated soil compared with the independent IMOs and n-HAP treatments. The combined treatment of γ-PGA and IMOs substantially increased shoot dry biomass in moderately contaminated soil. These results indicate that solo n-HAP enhanced plant growth and soil Cd and Pb immobilisation, and mitigated Cd and Pb accumulation in shoots. However, the combination of n-HAP and IMOs was optimal for stabilising and reducing HMs’ uptake and promoting plant growth in contaminated soil, suggesting its potential for safe crop production.

## 1. Introduction

Heavy metal (HM) pollution has long been a concern because of its limited biodegradability and toxicity to plants, animals, and humans [1,2,3]. The HMs, Pb and Cd, are ubiquitous pollutants in agricultural ecosystems owing to industrial practices, irrigation with sewage wastewater, application of livestock manure as fertiliser, and the use of agricultural chemicals [4,5]. Consequently, the remediation of HM-contaminated soils through soil replacement, chemical fixation, bioremediation, and plant stabilisation [6,7] is imperative for protecting the health and safety of the public and the environment [8].

Various in situ remediation techniques using various materials have been developed in recent decades [6,9]. Lime, phosphate, P-containing materials, clay minerals, metal oxides, and biochar have been widely used to decrease bioavailable HM in recent years [10,11]. Among them, apatite-based materials have been established to be effective in immobilising Pb, Cd, Cu, Zn, and other metals in soil [12]. Nanoscale phosphatic materials are particularly superior to their traditional bulk counterparts owing to their high adsorption capacity for HMs [13]. For example, nano-hydroxyapatite (n-HAP) is an ideal material for the disposal of contaminants because of its small particle size and increased surface area, which provide strong interactions with HMs [14,15,16]. For example, the concentration of Cd in potato tubers was reduced by 17.4% after applying n-HAP [17].

Poly γ-glutamic acid (γ-PGA) is a biological macromolecule formed from D-glutamic acid and L-glutamic acid monomers, which are joined via a gamma–glutamine bond [18,19,20]. The extracellular biopolymer, γ-PGA, is naturally occurring, being produced by microbes such as *Bacillus subtilis* and *Bacillus lichenformis* through fermentation [19]. Being a potential environment-friendly polymer with biodegradable, non-immunogenic, and non-toxic characteristics, γ-PGA can be used as a substrate to enhance the growth of microorganisms [19,21,22]. In recent years, γ-PGA has been widely applied in food processing [23], water treatment [24], agricultural industries [25], soil improvement, and the alleviation of abiotic stress in plants [26,27,28]. The numerous functional groups (–COOH) that γ-PGA contains can bind metal ions through a complex reaction. Previous studies have revealed that γ-PGA is effective for the adsorption of metal ions such as Ni^2+^, Cu^2+^, Zn^2+^, Cr^3+^, Hg^2+^, and U^4+^ during water treatment [29]. Several studies have reported the role of γ-PGA in alleviating HMs’ toxicity in plants. The effects of γ-PGA on cucumber seedlings were tested under Cd and Pb stress, demonstrating that γ-PGA effectively reduced the growth inhibitory effects of Cd and Pb on the plants, and the metal concentrations of the Cd + 1.2 g/L γ-PGA and Pb + 6 g/L γ-PGA groups were 38.65% and 74.13%, respectively, lower than those of the Cd and Pb only groups (i.e., the untreated groups) [30].

Compared with chemical sorbents, microorganisms are more environment-friendly because they attenuate the toxic influence of HMs on organisms without creating secondary pollution or destroying soil properties [31,32]. Microbial bioremediation can be achieved by utilising indigenous microorganisms (IMOs) in the soil environment to break down HMs into their benign components [33]. Compared with extraneous microbial communities, IMOs have greater adaptability and competitiveness within their specific soil environment [34]. Many studies have tested the natural remediation potential of IMOs on HM-polluted soils, and pronounced effects have been observed. For example, a study revealed that the total HM concentrations at several tailing sites decreased over 31 years of natural attenuation by IMOs, and the Cd and Zn concentrations decreased by 2.18 and 3.62 times, respectively [35].

Many studies have revealed that inoculation with microorganisms and amendments have synergistic effects in increasing soil fertility and plant performance in HM-contaminated soils [36,37]. The combination of biochar and arbuscular mycorrhizal fungi (AMF) inoculation could modify soil physicochemical properties, enhance AMF growth [38], and affect nutrient cycling [39]. Additionally, several studies have been conducted on the combined effects of n-HAP and microorganisms on the remediation of HM-polluted soils [10,40]. When combined with microorganisms, n-HAP improved the growth of plants and significantly decreased the uptake of HM. For example, Liu et al. revealed that, in Cd-contaminated soil, the combination of 0.5% (W/W) n-HAP with different concentrations of white rot fungi increased plant biomass by 17.95–23.93%, decreased plant Cd concentration by 38.16–40.94%, and significantly increased the activity of alkaline phosphatase and urease [41]. However, previous research has predominantly focused on a specific strain. ‘Soil ecological engineering’ has recently been proposed as an approach that combines management practices to enhance the overall biological diversity in human land-use systems with targeted manipulation of soil biota to improve agricultural sustainability [42]. Therefore, further confirmation is required to clarify whether IMOs combined with n-HAP and γ-PGA have a synergistic effect on HMs’ pollution remediation of agricultural land.

Some studies proved that people in Hunan and the other three provinces in southern China faced a high health risk of Pb and Cd when consuming vegetables [43]. *Coriandrum sativum* L. is an important vegetable crop that is widely used to flavour and garnish culinary dishes and is fundamental for supporting livelihoods [44]. In this study, we aimed to evaluate the independent and combined effects of IMOs with n-HAP and γ-PGA on plant growth, Pb and Cd uptake, and Pb and Cd immobilisation in soil. We hypothesised that the combined treatments would have an additive effect on soil alkalinisation and HMs’ stabilisation and modulate HMs’ uptake and coriander growth.

## 2. Materials and Methods

### 2.1. Soil and Amendments

Two Pb- and Cd-contaminated soil samples were collected from the topsoil layer (0–20 cm depth) of typical agricultural land in Zhuzhou City (27°50′2.9″ N, 113°25′9.5″ E), Hunan Province, China. The soil under investigation has been contaminated with HMs by wastewater discharge from a nearby metallurgy plant since the early 1980s. The soil type is red earths. The clay content of the soil is 21.7%.  Fifty kilograms of soil was collected from two sites with different concentrations of heavy metal. According to China’s soil environmental quality for agricultural land standard (GB15618-2018), the total Cd and Pb concentration in the two soils was below the soil screening value and below the intervention value, respectively (Table 1), thus the soils were regarded as having slightly and moderately contaminated levels. For both soils, a subsample of fresh soil was passed through a 2 mm sieve and stored at 4 °C for preparing the IMOs’ inoculum. The remaining soil was air-dried at around 25 degrees Celsius, ground, and passed through a 2 mm sieve and stored at room temperature. A portion of the soil was used as the substrate in the pot experiment, and the remainder was used for analysing the selected soil parameters. The physicochemical attributions of the two soils are presented in Table 1.

n-HAP and γ-PGA were purchased from the Nanjing Emperor Nano Material Co., Ltd. (Nanjing, China) and Yantai Jinbang Biotechnology Co., Ltd. (Yantai, China), respectively. n-HAP is an ultra-fine powder with a pH of 10.8, Ca/P molar ratio of 1.70, total HM content ≤ 1 mg/kg, and average particle size ≤ 60 µm. The molecular weight of γ-PGA used in this study is 1.0 × 10^6^ Da, and the density of aqueous solution is 1.11 g/mL. The mass concentration for the γ-PGA agents, which is produced from *B. subtilis*, is 4%. The Huoshan coriander (*Coriandrum sativum* L.) cultivar was selected for this experiment.

### 2.2. Pot Experiment

This experiment included three amendments, i.e., non-amendment control, n-HAP, and γ-PGA, and two microorganisms (non-inoculation control and IMOs). Two pollution levels were designed to represent slightly and moderately contaminated levels, respectively. A control treatment receiving no amendments was also included. Five replicates were performed for each treatment. The pots used in the experiment had a height of 18 cm and diameter of 16 cm and contained 1 kg of soil with or without amendments. The substrate soil was sterilized by γ-irradiation with 25 kGy ^60^Co. Two hundred grams of unsterilized IMOs and eight hundred grams of sterilized substrate soil were placed into the pot. n-HAP was added to the pots at a ratio of 1.5% each and homogeneously mixed according to the method of Zhu et al. (2019) [45], and the soil in each pot was then incubated for one week to equilibrate. γ-PGA was added to the pots at a ratio of 0.2% and homogeneously mixed, according to the method of Pang et al. (2018) [30], and the soils in each pot were then incubated for two weeks to equilibrate. Each pot was then sown with 20 pre-germinated seeds and thinned to six plants 15 days after emergence. The plants were grown in a glasshouse of China Agricultural University from April to July 2021, with a temperature of 20–25 °C, a relative humidity of 60–70%, and a light/darkness photoperiod of 14/10 h (light intensity, 240–350 μmol m^−2^ ·s^−1^). The pots were gravimetrically maintained at a water-holding capacity of approximately 60%, and the soil water holding capacity was determined by the ring knife method. No additional nutrients were applied in the experiments.

### 2.3. Harvest and Sample Analysis

#### 2.3.1. Plant Analysis

The plants were harvested 60 days after transplantation. After transporting to the laboratory, each plant sample was washed with deionised water, oven-dried at 100 °C to an even weight, and separated into roots and shoots to determine the dry weight of each component for the biomass growth measurements; then, a 50–100 g dry sample was collected for chemical analysis. The dried samples were pulverised using a stainless-steel grinder and then stored at room temperature. Dry plant material (0.25 g) was digested in 8 mL of HNO_3_ in a microwave oven (Start 1500, MLS GmbH, Leutkirch im Allgäu, Germany) using a temperature step gradient (maximum of 210 °C). When necessary, digested solutions were diluted with 2% HNO_3_. The Cd and Pb concentrations in the extract solution were measured via inductively coupled plasma mass spectrometry (ICP-MS 7700, Agilent Technologies, Santa Clara, CA, USA). Blanks and a certified reference material, GBW10045 (GSB-23, rice flour), were also prepared by the digestion process for quality assurance. The recovery of the reference material was between 87% and 110%.

#### 2.3.2. Soil Analysis

The soil from each pot was air-dried, ground by automatic agate mill, sieved into 2 mm, and mixed thoroughly; then, an around 200–300 g sample was collected for the analysis of metal availability and soil pH. The soil pH was measured in an aqueous soil suspension (1:2.5, *w*/*v*). The concentrations of available Cd and Pb were acquired by mechanically shaking each 3.0 g air-dried sample with 20 mL of 0.005 mol/L DTPA extract (0.005 mol/L DTPA + 0.01 mol/L CaCl_2_ + 0.1 mol/L triethanolamine, buffered to a pH of 7.30 using a hydrochloric acid solution or ammonia) [46]. After oscillation, the solution was centrifuged and immediately filtrated using a 0.45 μm filter membrane. Physical analysis of the filtrate was performed within 48 h via ICP-MS. Total Cd and Pb in substrate soil were digested with a solution of 3:1 HNO_3_/HClO_4_ (*v*/*v*), and the filtrate was also performed via ICP-MS.

### 2.4. Statistical Analysis

All data were tested for normality (Kolmogorov–Smirnov test) and homogeneity (Levene’s test) before statistical analysis. Data analyses were performed using the IBM SPSS statistical software package version 20 (IBM Corporation, New York, NY, USA) to obtain the significance and variance. The impact of three factors on plant growth, Cd and Pb concentration (in shoot and root), soil available HM concentration, and pH values was analysed by univariate analysis of variance (ANOVA) with the generalized linear model (GLM). Data were analysed using one-way ANOVA and Duncan’s multiple range test (*p* < 0.05) to compare the mean values for plant and soil data. All results are expressed as mean values and standard error (SE). All measurements were conducted in five replicates. Pearson correlation analysis was conducted to test the correlation among dry biomass; pH, Cd, and Pb concentration; and available Cd and Pb concentration at two contaminated levels.

## 3. Results

### 3.1. Plant Growth Responses

**Figure 1:**
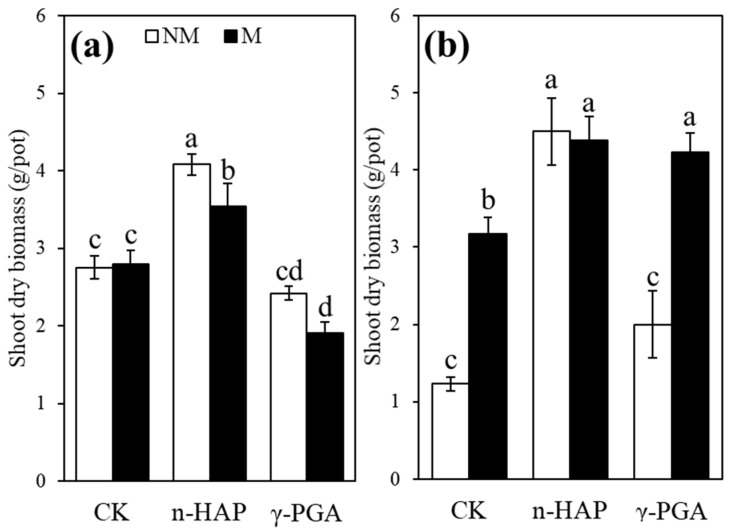
Shoot dry biomass of coriander (*Coriandrum sativum* L.) grown in slightly contaminated soil (**a**) and moderately contaminated soil (**b**). Non-inoculated control and indigenous microorganism (IMO) inoculation are represented by NM and M, respectively. Non-amendment control, nano-hydroxyapatite (n-HAP), and poly-γ-glutamic acid (γ-PGA) treatments are represented by CK, n-HAP, and γ-PGA, respectively. Data are represented as ±SE of five replicates. The significant differences between different treatments calculated using Duncan’s multiple range test following significant one-way ANOVA (*p* < 0.05) are indicated by different lowercase letters (a, b, c, d).

Shoot dry biomass was significantly (*p* < 0.05) affected by amendments, IMOs, contaminated levels, amendments × IMOs, amendments × contaminated levels, IMOs × contaminated levels, and amendments × IMOs × contaminated levels in contaminated soil, and root biomass was significantly (*p* < 0.05) affected by IMOs, contaminated levels, and their interaction (Table 2). The effects of IMOs and amendments on shoot biomass are presented in Figure 1a,b. Compared with the control treatment, regardless of amendments, independent treatment of moderately contaminated soil with IMOs increased shoot and root dry biomass by 2.58 and 2.92 times, respectively. Compared with that of the control treatment, the shoot dry biomass of coriander treated with n-HAP only increased by 1.48 and 3.66 times in slightly and moderately contaminated soils, respectively. Compared with the control group, when combined with the IMOs, the increase was 1.29 and 3.56 times in slightly and moderately contaminated groups, respectively. In moderately contaminated soil, the combined effect of γ-PGA and IMOs inoculation significantly (*p* < 0.05) increased shoot (3.44-fold) and root (5.42-fold) dry biomass compared with the control treatment. However, shoot dry biomass showed a slight decrease in γ-PGA in the IMOs group in the slightly contaminated soil. The addition of n-HAP and γ-PGA had almost no influence on root dry biomass in either soil type (Table 3).

### 3.2. Cd and Pb Uptake by Coriander Tissues

The shoot Cd and Pb concentrations and root Cd concentration were significantly (*p* < 0.05) affected by amendments and contaminated levels, whereas shoot Cd concentration was also significantly (*p* < 0.05) affected by IMOs and the interaction of IMOs and amendments. Root Pb concentration was significantly (*p* < 0.05) affected by amendments and the interaction of amendments and contaminated levels (Table 2).

**Figure 2:**
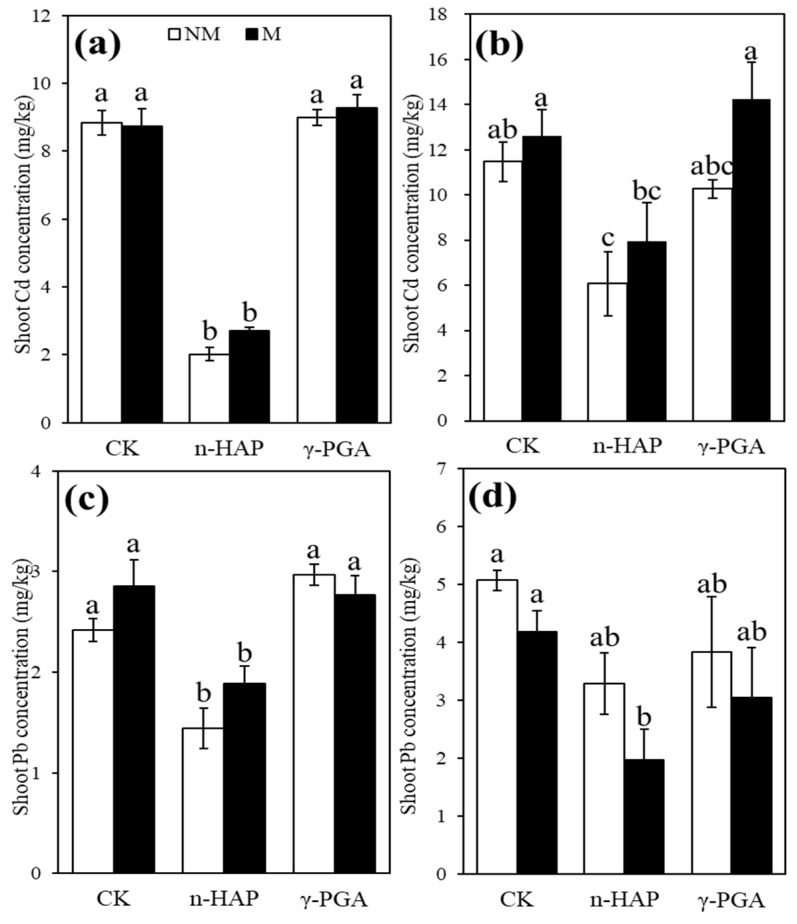
Shoot Cd concentrations (**a**,**b**) and Pb concentrations (**c**,**d**) of coriander in slightly contaminated soil (**a**,**c**) and moderately contaminated soil (**b**,**d**). Non-inoculated control and indigenous microorganism (IMO) inoculation are represented by NM and M, respectively. Non-amendment control, nano-hydroxyapatite (n-HAP), and poly-γ-glutamic acid (γ-PGA) treatments are represented by CK, n-HAP, and γ-PGA, respectively. Data are represented as ±SE of five replicates. The significant differences between different treatments calculated using Duncan’s multiple range test following significant one-way ANOVA (*p* < 0.05) are indicated by different lowercase letters (a, b, c).

The effects of inoculation and amendments on shoot Cd concentrations are shown in Figure 2a,b, respectively, and the shoot Pb concentrations are shown in Figure 2c,d. The application of n-HAP with and without IMOs significantly (*p* < 0.05) reduced shoot Cd and Pb concentrations in both soils (Figure 2). With the addition of n-HAP, shoot Cd concentrations reduced by 1.89 and 1.44 times in the non-inoculation control and IMOs groups, respectively, as compared with those in the controls in moderately contaminated soil. Compared with that of the control treatment, the Cd concentrations of the shoots from slightly contaminated soil decreased by 4.38 and 3.25 times in the non-inoculation control and IMOs groups, respectively. With the addition of n-HAP, the shoot Pb concentrations reduced by 1.68 and 1.28 times and root Pb concentrations reduced by 3.22 and 2.50 times in the non-inoculation control and inoculation treatments, respectively, as compared with those in the control treatment in slightly contaminated soil. In moderately contaminated soil, shoot and root Pb concentrations of the n-HAP and IMOs combined group reduced by 2.57 and 2.30 times, respectively, as compared with those of plants grown in the control group. However, rather than decreasing the Cd and Pb concentrations, γ-PGA approximately doubled the root Cd and Pb concentrations in slightly contaminated soil.

### 3.3. Soil Analysis

#### 3.3.1. Soil pH

**Figure 3:**
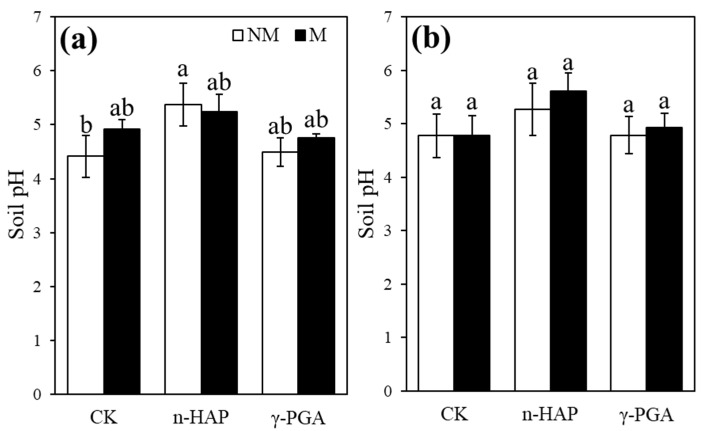
Soil pH following plant harvesting in slightly contaminated soil (**a**) and moderately contaminated soil (**b**). Non-inoculated control and indigenous microorganism (IMO) inoculation are represented by NM and M, respectively. Non-amendment control, nano-hydroxyapatite (n-HAP), and poly-γ-glutamic acid (γ-PGA) treatments are represented by CK, n-HAP, and γ-PGA, respectively. Data are represented as ±SE of five replicates. The significant differences between different treatments calculated using Duncan’s multiple range test following significant one-way ANOVA (*p* < 0.05) are indicated by different lowercase letters (a, b).

Soil pH was significantly (*p* < 0.05) affected by amendment (Table 2). Compared with the control treatment in slightly contaminated soil, the addition of n-HAP significantly increased soil pH by 0.96 and 0.83 in the non-inoculation control and inoculation treatments, respectively, but had no effect in moderately contaminated soil (Figure 3b). The addition of γ-PGA and inoculation with IMOs had almost no influence on the pH values in all treatment groups.

#### 3.3.2. Available Cd and Pb Concentrations

The available Cd and Pb concentration was significantly (*p* < 0.05) affected by amendments and contaminated levels. The available Cd concentration was also significantly (*p* < 0.05) affected by IMOs, IMOs × contaminated levels, and amendments × contaminated levels interactions (Table 2).

**Figure 4:**
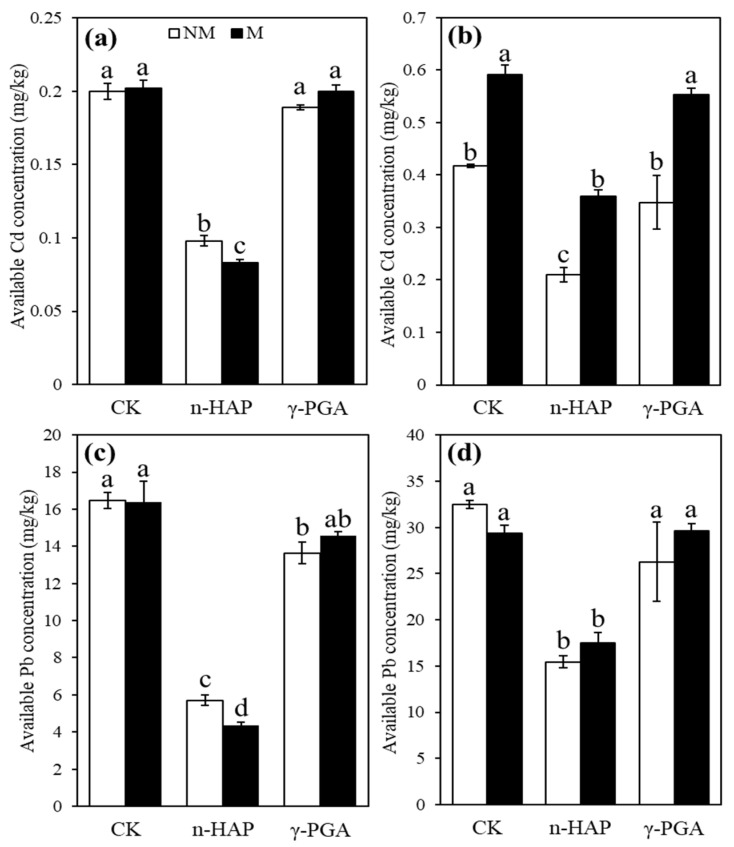
Available Cd concentration (**a**,**b**) and available Pb concentration (**c**,**d**) in slightly contaminated soil (**a**,**c**) and moderately contaminated soil (**b**,**d**). Non-inoculated control and indigenous microorganism (IMO) inoculation are represented by NM and M, respectively. Non-amendment control, nano-hydroxyapatite (n-HAP), and poly-γ-glutamic acid (γ-PGA) treatments are represented by CK, n-HAP, and γ-PGA, respectively. Data are represented as ±SE of five replicates. The significant differences between different treatments calculated using Duncan’s multiple range test following significant one-way ANOVA (*p* < 0.05) are indicated by different lowercase letters (a, b, c, d).

The effects of inoculation and amendments on the concentrations of available Cd are shown in Figure 4a. The available Pb concentrations are shown in Figure 4c. Regardless of amendments, IMOs independently increased the available Cd concentrations in moderately contaminated soil by 1.42 times compared with the control group. The application of n-HAP, with and without IMOs, significantly (*p* < 0.05) reduced the available Cd and Pb concentrations in both soils (Figure 4). Compared with the control group, when n-HAP was independently applied, available Cd concentrations reduced by 2.04 and 1.99 times and Pb concentrations reduced by 2.89 and 2.10 times in slightly and moderately contaminated soils, respectively. Compared with the control group, in the n-HAP and IMOs group combined, the available Cd concentrations decreased by 2.41 and 1.16 times and the available Pb concentrations decreased by 3.79 and 1.86 times in slightly and moderately contaminated soils, respectively. An additive effect was observed with n-HAP and IMOs reducing the available Cd and Pb concentrations in slightly contaminated soil to 0.08 mg/kg and 4.4 mg/kg, respectively. Compared with the control group, γ-PGA, with and without IMOs, exhibited inhibitory effects on available Pb concentrations in slightly contaminated soil, with a decrease of 1.13 and 1.21 times, respectively.

### 3.4. Correlation Analysis of Dry Biomass, Soil pH, Cd and Pb Concentrations, and Available Cd and Pb Concentrations

**Figure 5:**
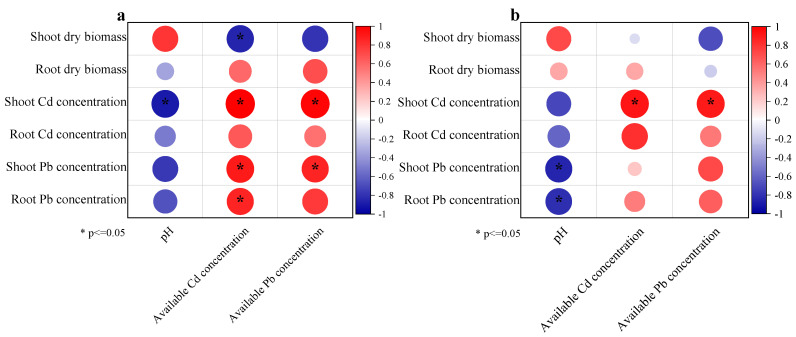
Correlation analysis among dry biomass, pH, Cd and Pb concentration, and available Cd and Pb concentration in slightly (**a**) and moderately (**b**) contaminated soils.

The correlation analysis results of shoot and root dry biomass, pH, Cd and Pb concentrations, and available Cd and Pb concentrations are shown in Figure 5. In the slightly contaminated soil, shoot dry biomass was negatively correlated with available Cd and shoot Pb concentrations, and Cd concentration was positively correlated with available Pb and Cd concentrations (*p* ≤ 0.05). Shoot Cd concentration was negatively correlated with soil pH. In moderately contaminated soil, shoot Cd concentration exhibited a positive correlation with available Pb and Cd concentrations, whereas shoot and root Pb concentrations were negatively correlated with soil pH.

## 4. Discussion

This study revealed a remarkable growth response and low Pb and Cd concentrations, which were beneficial to coriander, as a result of the (1) amendment with n-HAP, (2) amendment with γ-PGA, (3) inoculation with IMOs, (4) synergistic effect of IMOs and n-HAP in slightly contaminated soil, and (5) combined effect of γ-PGA and IMOs in moderately contaminated soil.

### 4.1. Effect of Pb and Cd Toxicity on Plant Growth

Shoot dry biomass was much higher in moderately contaminated soil than slightly contaminated soil, particularly in unsterilized treatments (Figure 1), which might imply the importance of IMOs in improving plant resistance to HM stress [47,48]. Previous research has also revealed that the stimulation of indigenous soil microbial activity is essential to enhance natural detoxification of contaminated environments [49].

In this study, growth inhibition of roots was observed in both soil treatment groups (Table 3). Growth inhibition in plants is a common physiological effect of HM stress. The toxicity varies between breeds and has an increased effect in sensitive plants [50]. Many key physiological processes, such as photosynthesis, respiration, reactive oxygen species (ROS) metabolism, and hormonal regulation, can be disrupted by HMs [51]; moreover, the life cycle of plants, from germination to final production, can be adversely affected [52,53]. The roots are the first components of a plant to be affected by stress conditions. Consequently, root length and viability are reduced, which eventually disrupt plant photosystems [54] and the absorption of essential nutrients [55]. In addition, root activity and biomass have been significantly decreased under soil acid stress [56].

### 4.2. Effect of n-HAP with and without IMOs

Overall, n-HAP decreased soil Pb and Cd bioavailability and Pb and Cd concentrations in plant shoots, which led to positive effects on plant growth. Simultaneously, soil acidity was mitigated by n-HAP application. These results correspond with those of previous studies. For example, the addition of n-HAP significantly decreased Cu and Zn uptake by ryegrass and increased its biomass [57]. These findings suggest that n-HAP has potential for the remediation of contaminated soil and safe production of crops. The immobilisation of HMs by n-HAP is primarily due to the formation of phosphate precipitates with a considerably low solubility and bioaccessibility [57,58,59,60]. Amendments with P-containing materials generally decrease Cd and Pb mobility by precipitating pyromorphite-type minerals [58,61,62,63]. In this study, shoot Pb and Cd concentrations decreased with n-HAP application, which is consistent with the low DTPA-extracted Cd and Pb concentrations in the soil.

Soil pH generally influences the mobility and bioavailability of metallic contaminants and their absorption by plants [64,65]. A high soil pH generally facilitates the immobilisation of Cd and Pb, leading to low phytoavailability [58,66]. Our results demonstrate that n-HAP increased the soil pH, and the soil pH correlated positively with the plant biomass, but negatively (*p* < 0.05) with shoot Cd and Pb concentrations (Figure 5), confirming that soil pH is a key factor influencing plant growth and controlling HMs’ bioavailability and accumulation by plants in acidic soil. Furthermore, the liming effect facilitates the precipitation of HMs by forming insoluble oxyhydroxides and hydroxides [67]. Owing to its chemical composition, n-HAP can release OH^−^ to increase soil pH, thereby inducing a liming effect [60]. Notably, in multiple-metal-contaminated soils, the presence of one metal can alleviate the immobilisation efficiency of the other because of adsorption site competition between them [67]. In this study, the available Pb concentration was positively related to shoot Cd concentration in both soils (Figure 5), thereby confirming this fact.

The combined application of IMOs and n-HAP exhibited synergistically decreased DTPA-extractable Cd and Pb concentrations in slightly contaminated soil; however, no synergistic effect was observed in moderately contaminated soil. Some studies have revealed that stabilising amendments in the heavy-metal-polluted soil aided the recovery of soil bacterial diversity and soil microbial metabolic activities related to Cd and Pb transformation and enriched genera related to nutrient activation, particularly at low metal concentrations [68,69,70,71]. Our results suggest that n-HAP, along with several taxa, may improve the immobilisation efficiencies of DTPA-extractable Cd and Pb in slightly contaminated soil, whereas IMOs from moderately contaminated soil developed many strains tolerant to HMs, thereby activating the available Cd in moderately contaminated soil.

### 4.3. Effect of γ-PGA with and without IMOs

In this study, growth inhibition was effectively alleviated in moderately contaminated soil and, in slightly contaminated soil, the available Pb was reduced when coriander was treated with γ-PGA. These results indicate that γ-PGA significantly alleviated the damage caused by HM stress. Similar results were obtained in previous studies, where the root and shoot biomass at Cd and Pb concentrations of exposed cucumber seedlings were significantly decreased, and growth inhibition was effectively alleviated following the addition of γ-PGA. This may be due to Cd^2+^ and Pb^2+^ adsorption by γ-PGA, whereby Cd and Pb transform from a free into a chelating state [30]. Several types of metal ions are effectively chelated by γ-PGA, and the reaction between γ-PGA and metals could change the helix–coil transition, which might affect the binding capacity of metals [72,73].

Previous research has revealed that HMs in soil reduce the amount of microbial biomass and change the community structure [74], and several studies have revealed that γ-PGA alleviates the toxic effects of Cd and Pb on soil microorganisms [30]. The effect of increasing shoot and root dry biomass in γ-PGA with IMO treatment in moderately contaminated soil was additive. Thus, γ-PGA can be used as a substrate to both improve the tolerance of some plant-growth-promoting microorganisms and increase the abundance of these microorganisms.

## 5. Conclusions

Applying n-HAP, with and without IMOs, significantly increased shoot dry biomass and reduced shoot Cd and Pb concentrations and DTPA-extracted Cd and Pb concentrations in both slightly and moderately contaminated soils in most cases. The application of γ-PGA, with and without IMOs, significantly reduced DTPA-extracted Pb in slightly contaminated soil. Irrespective of amendments, independently applied IMOs effectively increased shoot dry biomass and DTPA-extracted Cd in moderately contaminated soil. The combined application of IMOs with n-HAP synergistically decreased DTPA-extracted Cd and Pb in slightly contaminated soil, whereas the combination of γ-PGA and IMOs substantially increased shoot dry biomass in moderately contaminated soil. In conclusion, these results indicated that n-HAP can effectively improve crop production and decrease the Cd and Pb concentrations in coriander cultivated in soils contaminated by HMs, while the effect of γ-PGA was limited. However, the combination of n-HAP and IMOs only had a synergistically effect in reducing HMs’ availability at slightly contaminated levels, while γ-PGA with IMOs only showed an additive effect in promoting plant growth at moderately contaminated levels. Therefore, if microorganisms are properly controlled, soil amendments have a wide application potential for controlling HMs’ pollution of agricultural land, thereby improving crop growth. In addition, more appropriate soil amendments should be selected for safe crop production according to HMs’ pollution levels in the contaminated soil.

## Figures and Tables

**Table 1:** Major physicochemical attributions of the contaminated soil.

Soils	pH	EC	TP	Olsen-P	SOC	Available Cd	Total Cd	Available Pb	Total Pb
		(ds/cm)	(g/kg)	(mg/kg)	(g/kg)	(mg/kg)	(mg/kg)	(mg/kg)	(mg/kg)
Slightly contaminated	4.56 ± 0.25	376 ± 7	0.50 ± 0.10	17.9 ± 0.5	26.5 ± 1.1	0.21 ± 0.01	0.29 ± 0.01 (0.30) ^1^	13.8 ± 0.5	50.9 ± 3.0 (70.0) ^1^
Moderately contaminated	3.85 ± 0.13	271 ± 4	0.82 ± 0.03	60.0 ± 2.9	17.0 ± 0.3	0.66 ± 0.06	1.08± 0.05 (1.50) ^2^	29.4 ± 1.0	100.0 ± 1.8 (400.0) ^2^

Note: ^1, 2^ Data are from the “Soil Environmental Quality—Risk control standard for soil contamination of agricultural land (GB 15618-2018)”. ^1^ “Screening value” means potential risks for agricultural food security, crop growth, or soil quality if pollutant concentrations exceed this value. ^2^ “Intervention value” means the soil should be strictly controlled for agricultural production if pollutant concentrations exceed this value. Abbreviations: EC, electrical conductivity; TP, total phosphorous; Olsen-P, available phosphorous; SOC, soil organic carbon. Data are mean ± SE (n = 3).

**Table 2:** Significance levels (*F* value) of indigenous microorganism, amendments, contaminated levels and their second- and third-order interactions in shoot biomass, shoot Cd and Pb concentration, root biomass, root Cd and Pb concentration, available Cd and Pb concentration, and pH values according to a three-way ANOVA.

Parameter	M	A	CL	M × A	M × CL	A × CL	M × A × CL
Shoot biomass	13.3 ***	56.8 ***	5.9 *	9.1 ***	37.1 ***	11.1 ***	6.0 **
Shoot Cd concentration	7.0 *	54.1 ***	63.1 ***	4.2 *	0.9	1.0	0.7
Shoot Pb concentration	2.4	12.5 ***	22.6 ***	0.1	6.1 *	2.9	0.5
Root biomass	6.1 *	0.4	20.0 ***	1.4	5.5*	1.7	1.7
Root Cd concentration	4.0	4.1 *	30.8 ***	2.5	2.1	1.6	2.1
Root Pb concentration	0.6	20.6 ***	0.8	2.7	1.3	6.0 **	1.2
pH values	1.0	5.0 *	0.7	0.1	0.0	0.0	0.5
Available Cd concentration	98.2 ***	128.0 ***	804.0 ***	1.8	99.7 ***	12.6 ***	0.3
Available Pb concentration	0.1	107.0 ***	310.0 ***	2.2	0.4	1.5	1.8

Note: M represents the two inocula (non-inoculated control and indigenous microorganism); A represents the amendments (CK, n-HAP, and γ-PGA); and CL represents the contaminated levels (slightly and moderately contaminated). The significance of the effect is indicated by * *p* < 0.05, ** *p* < 0.01, and *** *p* < 0.001.

**Table 3:** Dry biomass, Cd concentration, and Pb concentration in the root of coriander (*Coriandrum sativum* L.).

Treatments	Dry Biomass (g/pot)		Cd Concentration (mg/kg)		Pb Concentration (mg/kg)	
	NM	M	NM	M	NM	M
Slightly contaminated						
CK	0.064 a	0.082 a	3.18 cd	4.69 bc	23.8 b	38.9 a
n-HAP	0.040 a	0.042 a	2.47 d	3.25 cd	7.4 c	9.51 c
γ-PGA	0.050 a	0.042 a	6.04 ab	6.73 a	47.1 a	49.8 a
Moderately contaminated						
CK	0.065 b	0.190 ab	9.62 b	86.0 a	34.5 ab	40.7 a
n-HAP	0.200 ab	0.225 ab	10.2 b	7.6 b	33.4 ab	15.0 b
γ-PGA	0.078 b	0.353 a	11.0 ab	18.7 ab	31.7 ab	40.0 a

Note: NM, non-inoculated control; M, indigenous microorganisms; CK, non-amendment control; n-HAP, nano-hydroxyapatite; γ-PGA, poly-γ-glutamic acid. Data are represented as mean of five replicates; different letters indicate significant differences between six treatments at *p* < 0.05.

## Data Availability

Not applicable.
